# The Long Noncoding RNA Hotair Regulates Oxidative Stress and Cardiac Myocyte Apoptosis during Ischemia-Reperfusion Injury

**DOI:** 10.1155/2020/1645249

**Published:** 2020-03-12

**Authors:** Kai Meng, Jiao Jiao, Rui-Rui Zhu, Bo-Yuan Wang, Xiao-Bo Mao, Yu-Cheng Zhong, Zheng-Feng Zhu, Kun-Wu Yu, Yan Ding, Wen-Bin Xu, Jian Yu, Qiu-Tang Zeng, Yu-Dong Peng

**Affiliations:** Department of Cardiology, Union Hospital, Tongji Medical College, Huazhong University of Science and Technology, Wuhan 430022, China

## Abstract

Oxidative stress and subsequent cardiac myocyte apoptosis play central roles in the initiation and progression of myocardial ischemia-reperfusion (I/R) injury. Homeobox transcript antisense intergenic RNA (*Hotair*) was previously implicated in various heart diseases, yet its role in myocardial I/R injury has not been clearly demonstrated. Mice with cardiac-restricted knockdown or overexpression of *Hotair* were exposed to I/R surgery. H9c2 cells were cultured and subjected to hypoxia/reoxygenation (H/R) stimulation to further verify the role and underlying mechanisms of *Hotair* in vitro. Histological examination, molecular detection, and functional parameters were determined in vivo and in vitro. In response to I/R or H/R treatment, *Hotair* expression was increased in a bromodomain-containing protein 4-dependent manner. Cardiac-restricted knockdown of *Hotair* exacerbated, whereas *Hotair* overexpression prevented I/R-induced oxidative stress, cardiac myocyte apoptosis, and cardiac dysfunction. Mechanistically, we observed that *Hotair* exerted its beneficial effects via activating AMP-activated protein kinase alpha (AMPK*α*). Further detection revealed that *Hotair* activated AMPK*α* through regulating the enhancer of zeste homolog 2/microRNA-451/calcium-binding protein 39 (EZH2/*miR-451*/Cab39) axis. We provide the evidence that endogenous lncRNA *Hotair* is an essential negative regulator for oxidative stress and cardiac myocyte apoptosis in myocardial I/R injury, which is dependent on AMPK*α* activation via the EZH2/*miR-451*/Cab39 axis.

## 1. Introduction

Acute myocardial infarction (AMI) is the leading cause of death worldwide due to the induction of congestive heart failure and life-threatening arrhythmias [1]. The survival and long-term prognosis in patients after AMI largely depend on the extent of cardiac myocyte death in ischemic myocardium. Percutaneous coronary intervention (PCI) is an effective therapeutic intervention to restore coronary blood flow and preserve the viable cardiac myocytes [2]. However, revascularization by PCI can cause additional ischemia-reperfusion (I/R) damage, which hampers myocardial salvage and diminishes the maximum potential benefit of this intervention [3]. Therefore, an extensive dissection about the molecular basis for myocardial I/R injury and the identification of novel therapeutic targets are scientifically and clinically important.

Despite multiple events, including inflammation, endoplasmic reticulum stress, autophagy, and intracellular Ca^2+^ overload, have been reported to be involved in the pathogenesis of I/R injury, emerging evidences suggest that oxidative stress and subsequent cardiac myocyte apoptosis play central roles in the initiation and progression of myocardial I/R injury [4, 5]. Mitochondria are the major source of reactive species oxygen (ROS) within the myocardium [6]. However, their normal structure and physiologic function are markedly compromised during cardiac ischemia, which then drive excessive ROS generation and elicit oxidative damage after the rapid restoration of coronary perfusion [7]. In addition, the expression of antioxidant enzymes in murine hearts is notably downregulated by I/R injury, which further aggravates I/R-triggered oxidative damage to the heart. Unstrained ROS accumulation induces oxidative damage to biological macromolecules, including DNA, lipids, and proteins, eventually resulting in cell apoptosis. Conversely, suppression of ROS overproduction significantly prevents I/R-induced cardiac myocyte apoptosis and cardiac dysfunction [8, 9]. AMP-activated protein kinase alpha (AMPK*α*), a highly conserved eukaryotic serine/threonine protein kinase, is proved to have beneficial effects in multiple cardiac pathophysiological conditions, including endoplasmic reticulum stress, energetic homeostasis, cellular calcium handling, cardiac hypertrophy, and fibrogenesis [10–13]. Russell et al. previously verified that AMPK*α* was responsible for the activation of glucose uptake and glycolysis during I/R-injured hearts, and prevented I/R-induced cardiac myocyte damage [14]. Besides, AMPK*α* was also implicated in regulating oxidative stress and cell survival in cardiac diseases [8, 15]. Wang et al. recently found that AMPK*α* overexpression could provoke efficient mitophagy to eliminate damaged mitochondria, thereby preventing ROS overproduction and cardiac myocyte apoptosis in the development of heart failure [16]. Moreover, numerous studies determined that AMPK*α* activation could attenuate myocardial I/R injury via blocking oxidative damage and cell apoptosis, whereas AMPK*α*-deficient mice exhibited increased ROS generation and cell apoptosis in response to myocardial I/R injury [15, 17, 18]. Therefore, targeting AMPK*α* may be of great therapeutic interest for treating I/R-induced injury.

Long noncoding RNAs (lncRNAs) are identified as kinds of non-protein-coding RNAs with the length longer than 200 nucleotides. Despite initially considered as the nonfunctional byproducts of RNA polymerase II transcripts, lncRNAs are now verified to participate in regulating numerous pathophysiological processes, ranging from cell proliferation, differentiation, senescence, to cell death [19, 20]. Recently, lncRNAs have attacked increasing attentions for their critical roles in the progression of cardiovascular diseases, especially myocardial I/R injury [21]. Homeobox transcript antisense intergenic RNA (*Hotair*) is a 2148 nucleotide lncRNA located in the HOXC cluster on chromosome 12 in humans [22, 23]. Previous studies showed that *Hotair* was implicated in the pathogenesis of various heart diseases, such as cardiac hypertrophy, myocardial infarction, diabetic cardiomyopathy, and sepsis-related cardiac injury [24–27]. Data from Lai et al. indicated that *Hotair* overexpression reduced cell surface area and the expression of hypertrophic markers via sponging microRNA-19 (*miR-19*) [25]. In a septic mouse model, Wu et al. found that *Hotair* was significantly upregulated in cardiomyocytes from septic mice, and *Hotair* silence reduced inflammatory response and improved sepsis-related cardiac dysfunction [26]. Consistently, *Hotair* expression was found to be increased in heart tissues subjected to AMI surgery, which then promoted myocardial inflammation and malfunction after AMI [27]. In contrast, results from Zhang lab verified *Hotair* overexpression alleviated AMI or hypoxia-induced inflammation and cardiac myocyte apoptosis [28]. And a recent study by Gao et al. further confirmed that *Hotair* knockdown increased, whereas cardiomyocyte-specific *Hotair* overexpression decreased inflammation, oxidative stress, and cardiac myocyte death in diabetic mice [24]. However, the role and potential molecular basis of *Hotair* in myocardial I/R injury have not been clearly clarified. The complex function of *Hotair* in cardiac diseases prompted us to investigate its credible role in myocardial I/R injury.

## 2. Materials and Methods

### 2.1. Animals and Treatments

Male C57BL/6 mice (6-10 weeks; 22-27 g) were obtained from HFK Bioscience Co., Ltd. (Beijing, China) and were bred in a specific pathogen-free laboratory environment at the Animal Center of Tongji Medical College of Huazhong University of Science and Technology. All animal experiments were consistent with the principles of the Care and Use of Laboratory Animals (NIH publication no. 85–23, revised 1996), which were also approved by the Animal Care and Use Committee of the Union Hospital of Huazhong University of Science and Technology. Cardiac-restricted knockdown or overexpression of *Hotair* was achieved by the adeno-associated virus 9 (AAV9) system as previously described [29, 30]. Briefly, mice were injected with AAV9 carrying *Hotair* (AAV9-*Hotair*, *Hotair*) or short hairpin RNA against *Hotair* (AAV9-sh*Hotair*, sh*Hotair*) under the cTnT promoter via the tail vein at a dose of 1 × 10^11^ viral genome particles per mice 4 weeks before I/R surgery to overexpress or knock down *Hotair* in the myocardium, respectively, whereas the mice assigned to the control group were injected with AAV9-*Ctrl* (*Ctrl*) or AAV9-sh*Ctrl* (sh*Ctrl*). The *Hotair* full genome (NR_003716.3) was cloned into pAAV-cTNT-MCS-ZsGreen carrier and amplified with the Stbl3 Escherichia coli to generate AAV9-*Hotair* virus. The AAV9-sh*Hotair* was cloned from the interfering sequence of *Hotair* (#n397142, Thermo Fisher Scientific).

**Figure 1:**
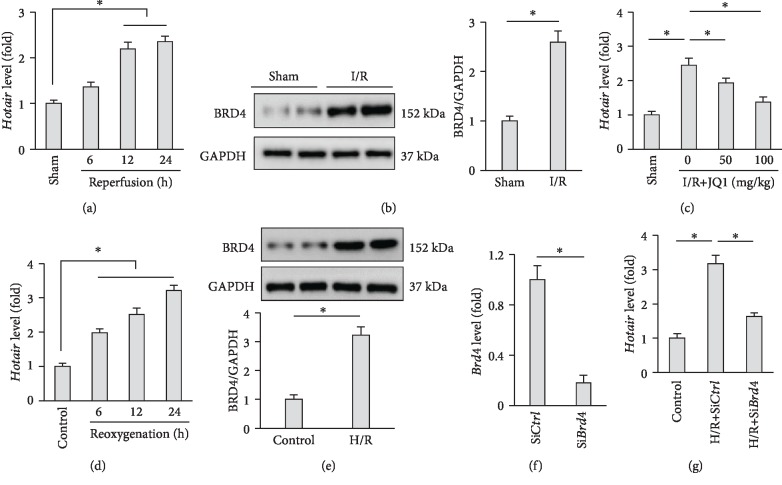
*Hotair* expression is increased by BRD4 in response to I/R stimulation. (a) *Hotair* expression within the reperfused myocardium in the indicated time points (*n* = 8). (b) Western blot images and the quantitative data of BRD4 in hearts subjected to ischemia for 30 minutes and reperfusion for additional 24 hours (I/R) (*n* = 6). (c) *Hotair* expression in murine hearts after I/R injury with or without JQ1 treatment (*n* = 8). (d) *Hotair* expression in H9c2 cells subjected to hypoxia for 2 hours and reoxygenation for the indicated time points (*n* = 8). (e) Western blot images and the quantitative data of BRD4 in H9c2 cells subjected to hypoxia for 2 hours and reoxygenation for additional 24 hours (H/R) (*n* = 6). (f) The efficiency of small interfering RNA against BRD4 (si*Brd4*) detected by PCR data (*n* = 8). (g) *Hotair* expression in H9c2 cells after H/R stimulation with or without si*Brd4* (*n* = 8). Data are presented as mean ± standard deviation (SD). ^∗^*P* < 0.05 versus the matched group.

Myocardial I/R injury mouse model was generated as previously described [4, 9]. Briefly, mice were injected with pentobarbital sodium (50 mg/kg, i.p.) and ventilated via intubation for anesthetization. Murine hearts were exposed by a left thoracotomy incision and then a slipknot was made around the left anterior descending coronary artery (LAD) against a PE10 tubing by an 8-0 Prolene suture. Animals assigned to sham-operated groups received the same procedure, except the snare was left untied. After 30 minute ligation, the occlusion was released to allow reperfusion for additional 24 hours except for specific annotations in Figure 1. During the surgical processes, the mice were kept warm on the heating pad. To verify the role of AMPK*α*, the mice were pretreated with compound C (CpC, 20 mg/kg, every other day) for 2 weeks before I/R surgery [11]. In addition, the mice were pretreated with JQ1 intraperitoneally at the indicated dosage 24 hours prior to I/R surgery to clarify the role of bromodomain-containing protein 4 (BRD4) in vivo.

### 2.2. Echocardiography Measurements

Transthoracic echocardiography was performed to measure left ventricle ejection fraction (LVEF) and left ventricle end-systolic dimension (LVESD) using the Vevo 1100 ultrasound system (Visual Sonics, Toronto, Canada) equipped with 30 MHz linear transducer according to our previous study [31]. Briefly, the mice were anesthetized by 2% isoflurane and then were exposed to the two-dimensional (2D) imaging echocardiography at the parasternal short axis to observe papillary muscles and septal wall. After that, M-mode imaging was recorded to measure the cardiac morphology and contractility. Echocardiography images were analyzed using the echo work station, and the functional parameters were calculated and averaged from six continuous cardiac cycles. All data acquisition and subsequent analysis were performed in a blinded manner.

### 2.3. Determination on the Size of Area at Risk (AAR) and Infarction Area (IA)

After reperfusion for 24 hours, the left coronary artery was reoccluded at the same position, and then 1% Evans blue was injected into the left ventricular chamber to calculate the AAR. Next, murine hearts were quickly excised, frozen at -20°C for 30 minutes, and subsequently cut into 5 short-axis slices, which were incubated in 1% 2,3,5-triphenyltetrazolium chloride (TTC, Sigma) at 37°C for 15 minutes to delineate the infarcted myocardium. All slices were photographed by a digital camera (Nikon Corp., Tokyo, Japan), and AAR (nonblue, including viable myocardium and infarcted area) together with IA (pale) was quantified by computerized planimetry using ImageJ software (NIH, Bethesda, MD, USA) [4, 9]. The AAR was expressed as percent of the total left ventricular area, whereas IA was expressed as percent of the AAR.

### 2.4. Western Blot Analysis

Total proteins were isolated from cultured cells or freshly removed left ventricles as previously described [32]. Briefly, the myocardial homogenates or cell lysates were prepared in a RIPA lysis buffer with protease inhibitor cocktail (Roche), which were then subjected to scraping, sonication, and centrifugation. After that, the protein concentration was determined by bicinchoninic acid (BCA) reagents (Pierce biotechnology, Rockford, IL), and equal amounts of protein samples were separated by sodium dodecyl sulfate polyacrylamide gel electrophoresis (SDS-PAGE). Next, the proteins were transferred to polyvinylidene fluoride (PVDF; EMD Millipore, USA) membranes, followed by the incubation with 5% skim milk at room temperature for 0.5 hour and the indicated antibodies at 4°C overnight. Next day, the membranes were incubated with the secondary antibodies at room temperature for 1.5 hours and then were scanned by the ChemiDoc™ XRS+ System (Bio-Rad Laboratories, Inc.). The primary antibodies for western blot analysis were provided as follows: BRD4 (Abcam; #ab128874, 1 : 1000 dilution), glyceraldehyde-3-phosphate dehydrogenase (GAPDH; Cell Signaling Technology; #2118, 1 : 3000 dilution), Mn-superoxide dismutase (MnSOD; Abcam; #ab13533, 1 : 1000 dilution), catalase (CAT; Abcam; #ab52477, 1 : 1000 dilution), B cell lymphoma-2 (BCL-2; Abcam; #ab59348, 1 : 1000 dilution), BCL-2 associated X (BAX; Cell Signaling Technology; #2772, 1 : 1000 dilution), NADPH oxidase 2 (NOX2; Abcam; #ab129068, 1 : 1000 dilution) phosphorylated protein kinase B (p-AKT; Cell Signaling Technology; #4060, 1 : 1000 dilution), total-AKT (t-AKT; Cell Signaling Technology; #2920, 1 : 1000 dilution), sirtuin1 (SIRT1; Abcam; #ab110304; 1 : 1000 dilution), p-AMPK*α* (Cell Signaling Technology; #2535, 1 : 1000 dilution), t-AMPK*α* (Cell Signaling Technology; #5831, 1 : 1000 dilution), p-acetyl coenzyme A carboxylase (p-ACC; Cell Signaling Technology; #3661, 1 : 1000 dilution), t-ACC (Cell Signaling Technology; #3676, 1 : 1000 dilution), calcium-binding protein 39 (Cab39; Abcam; #ab51132, 1 : 1000 dilution), and zeste homolog 2 (EZH2; Cell Signaling Technology; #5246; 1 : 1000 dilution).

### 2.5. RNA Extraction and Transcript Analysis

Total RNA was extracted from heart tissue or cultured cells by TRIZOL reagent (Takara Biotechnology, Dalian, China) and converted to cDNA using the PrimeScript RT reagent kit (Takara Biotechnology, Dalian, China). Quantitative real-time PCR was performed using the ABI PRISM 7900 Sequence Detector system (Applied Biosystems, Foster City, CA). Gene expressions were calculated using the *ΔΔ*Ct methods. The relative mRNA levels were normalized to GAPDH, whereas relative lncRNA or miRNA levels were normalized to the U6 expression according to previous studies [31, 32].

### 2.6. Measurement of Cardiac Troponin I (cTnI), N-Terminal B-Type Natriuretic Peptide (NT-proBNP), and Creatine Kinase (CK) in Serum

Serum levels of cTnI, NT-proBNP, and CK were measured by an automatic biochemical analyzer (Cobas 8000, Roche) as previously described [33].

### 2.7. Cell Culture and Treatments

H9c2 cells were purchased from ATCC (Manassas, VA, USA) and cultured in Dulbecco's modified Eagle's medium (DMEM) containing 10% fetal bovine serum (FBS). To imitate the I/R injury in vitro, H9c2 cells were preincubated in Krebs buffer with pyruvate (5 *μ*M) and sodium sulfite (50 *μ*M) in the humidified incubator (5% CO_2_/95% N_2_, 37°C) for 2 hours, which were then replaced with fresh DMEM containing 10% FBS under normoxic conditions (5% CO_2_/95% air, 37°C) for additional 24 hours to generate hypoxia/reoxygenation (H/R) cell model according to previous studies with a little modifications [34, 35]. To notify the role of BRD4 in vitro, H9c2 cells were incubated with small interfering RNA against BRD4 (si*Brd4*, 50 nM; #RSS338226, Thermo Fisher Scientific) using Lipofectamine RNAiMAX (Invitrogen) for 24 hours before H/R stimulation [32]. To knock down the expression of *Hotair* in vitro, H9c2 cells were infected with adenovirus carrying sh*Hotair* (Adsh*Hotair*) at the multiplicity of infection (MOI) of 150 in serum-free DMEM medium for 4 hours or Adsh*Ctrl* as the control. Cells were preinfected with adenovirus carrying *Hotair* (Ad*Hotair*, MOI = 60) for 4 hours to overexpress *Hotair* in H9c2 cells or Ad*Ctrl* as the negative control. After adenoviral delivery experiments, the cells were cultured in normal medium for additional 48 hours before the H/R stimulation. To inhibit AMPK*α* in vitro, cells were preincubated with CpC (10 *μ*M) for 30 minutes before the adenoviral infection [34]. To verify the involvement of *miR-451* and EZH2, cells were pretreated with *miR-451* inhibitor (50 nM; #AM17000, Thermo Fisher Scientific) or si*Ezh2* (50 nM; #RSS319678, Thermo Fisher Scientific) using Lipofectamine RNAiMAX (Invitrogen) for 24 hours.

### 2.8. Biochemical Analysis

The levels of malondialdehyde (MDA), 4-hydroxynonenal (4-HNE) and 3-nitrotyrosine (3-NT) in freshly removed left ventricles and cultured cells were detected by the commercial kits (Abcam, UK) according to the instructions [33]. Protein carbonylations (PCs) were measured spectrophotometrically at 360 nm as previously described [36]. Enzymatic activities of superoxide dismutase (SOD) and catalase (CAT) were detected using the commercial kits (Nanjing Jiancheng Bioengineering Institute, China) by a microplate reader (Synergy HT, BioTek, USA) according to the manufacturer's instructions.

### 2.9. Assessment of Cardiac Myocyte Apoptosis

Terminal deoxynucleotidyl transferase-mediated dUTP nick end-labeling (TUNEL) staining was performed to detect cell apoptosis in vivo and in vitro using a commercial kit (Millipore, USA) as previously described [37]. To quantify the apoptotic cells in the myocardium, more than 15 high power fields (×400 magnification) per heart were included in a blinded manner. Apoptotic index was calculated as the ration between TUNEL-positive cardiac myocyte nuclei and the total number of nuclei. Cell counting kit-8 (CCK-8) was used to further determine cell viability in vitro [32]. Moreover, supernatants derived from the cell lysates of murine hearts or cultured cells were collected for the assessment of caspase3 activity by using peptide-based DEVD-pNA as substrate [38].

### 2.10. 2′,7′-Dichlorodihydrofluorescein Diacetate (DCFH-DA) Staining

DCFH-DA staining was performed to evaluate ROS generation in H9c2 cells via referring to previous studies [39]. In brief, cells were incubated with DMEM medium containing 10 *μ*mol/L DCFH-DA (Beyotime Biotechnology, China) for half an hour at 37°C, and then the images were captured by an Olympus IX53 fluorescence microscope in a blinded manner.

### 2.11. Statistical Analysis

Results were shown as mean ± standard deviation (SD). Comparisons between two groups were performed using two-tailed Student's *t*-test, whereas multigroup comparisons were performed by one-way ANOVA analysis (SPSS 22.0). A *P* value less than 0.05 was considered statistically significant.

## 3. Results

### 3.1. Hotair Expression Is Increased by BRD4 in Response to I/R Stimulation

To explore the possible role of *Hotair* in myocardial I/R injury, we first detected the expression of *Hotair* in response to I/R injury in murine hearts. As shown in Figure 1(a), myocardial *Hotair* expression was significantly upregulated during heart reperfusion. Then, we investigated the underlying mechanism for the alteration of *Hotair* expression during I/R injury. Proteins of BRD family are epigenetic modulators that have been identified to play critical roles in regulating cardiovascular diseases and oxidative stress [40, 41]. Previous studies verified that BRD4 was abundantly expressed within the myocardium and it could directly bind to the *Hotair* promoter, thereby promoting *Hotair* expression in glioblastoma cells [42]. Intriguingly, we found that BRD4 protein level was also increased by myocardial I/R injury, and the inhibition of BRD4 by JQ1 notably abolished I/R-induced upregulation of *Hotair* in murine hearts (Figures 1(b) and 1(c)). To further confirm the observation, we established H/R models in H9c2 cells, and the data proved that *Hotair* expression together with BRD4 protein level were both increased by H/R stimulation (Figures 1(d) and 1(e)). BRD4 knockdown markedly decreased *Hotair* expression in H9c2 cells after H/R stimulation (Figures 1(f) and 1(g)). Collectively, these data demonstrated that *Hotair* expression was increased by BRD4 in response to I/R stimulation, suggesting that *Hotair* might be implicated in the pathogenesis of myocardial I/R injury.

### 3.2. Hotair Prevents Myocardial Injury Caused by I/R

**Figure 2:**
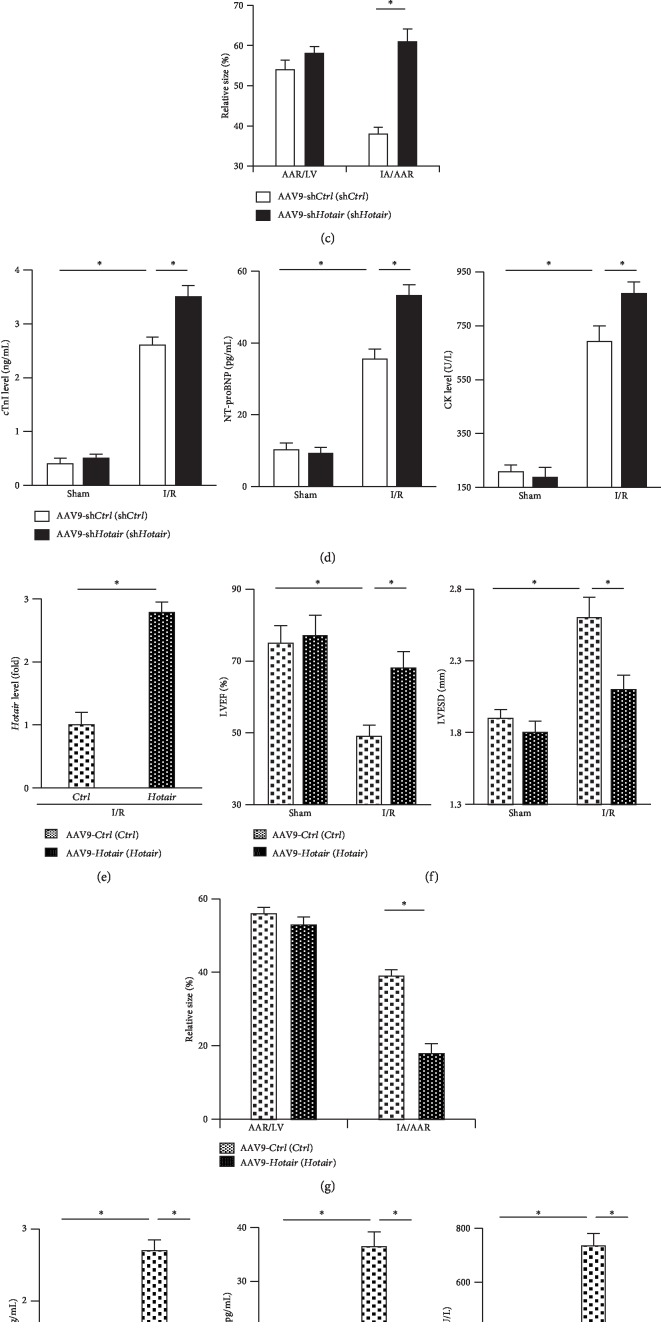
*Hotair* prevents myocardial injury caused by I/R. (a) *Hotair* expression in murine hearts in the indicated groups (*n* = 8). (b) Left ventricular ejection fraction (LVEF) and end-systolic diameter (LVESD) in mice subjected to I/R injury with or without *Hotair* knockdown (*n* = 10). (c) Morphometric analysis of the size of the area at risk (AAR) and infarction area (IA) in I/R-treated mice injected with AAV9-sh*Ctrl* or AAV9-sh*Hotair* (*n* = 8). (d) Serum biomarkers related to cardiac injury in mice subjected to I/R injury with or without *Hotair* knockdown (*n* = 8). (e) The efficiency of *Hotair* overexpression in I/R-injured murine hearts by AAV9-*Hotair* (*n* = 8). (f) Functional parameters in mice subjected to I/R injury with or without *Hotair* overexpression (*n* = 10). (g) Morphometric analysis of AAR and IA in I/R-treated murine hearts with or without AAV9-*Hotair* treatment (*n* = 8). (h) Serum biomarkers related to cardiac injury in mice subjected to I/R injury with or without *Hotair* overexpression (*n* = 8). Data are presented as mean ± SD. ^∗^*P* < 0.05 versus the matched group.

To examine the role of endogenous *Hotair* in I/R-mediated myocardial injury and dysfunction, we knocked down *Hotair* expression in murine hearts via the AAV9 system. As shown in Figure 2(a), *Hotair* expression within the I/R-injured myocardium was attenuated by sh*Hotair* injection. In response to myocardial I/R injury, *Hotair*-insufficient mice exhibited markedly decreased LVEF and elevated LVESD compared with that in control groups (Figure 2(b)). Furthermore, *Hotair* knockdown significantly increased the ischemia area (IA) without affecting the size of area at risk (AAR) after I/R, as evidenced by the AAR/LV and IA/AAR (Figure 2(c)). In addition, serum levels of biomarkers related to myocardial injury, including cTnI, NT-proBNP, and CK, were further elevated in mice after *Hotair* knockdown (Figure 2(d)). Collectively, these data indicated that *Hotair* knockdown exacerbated I/R-induced myocardial injury and the resultant heart dysfunction.

To further clarify the role of *Hotair*, mice were injected with AAV9-*Hotair* to overexpress *Hotair* in murine hearts or AAV9-*Ctrl* as the negative control 4 weeks prior to I/R surgery, and *Hotair* overexpression within the I/R-injured myocardium was validated in Figure 2(e). As shown in Figures 2(f) and 2(g), mice with *Hotair* overexpression exhibited a notable alleviation of cardiac dysfunction and the size of ischemia area was also reduced. Consistently, serum levels of myocardial injury biomarkers were also drastically suppressed in *Hotair*-overexpressed mice (Figure 2(h)). Interestingly, neither *Hotair* overexpression nor knockdown markedly affected the cardiac function under basal conditions compared with their matched controls. Taken together, these gain- and loss-of-function studies verified that *Hotair* was essential for the pathogenesis of I/R-induced myocardial injury.

### 3.3. Hotair Protects against I/R-Induced Oxidative Stress and Cardiac Myocyte Apoptosis

**Figure 3:**
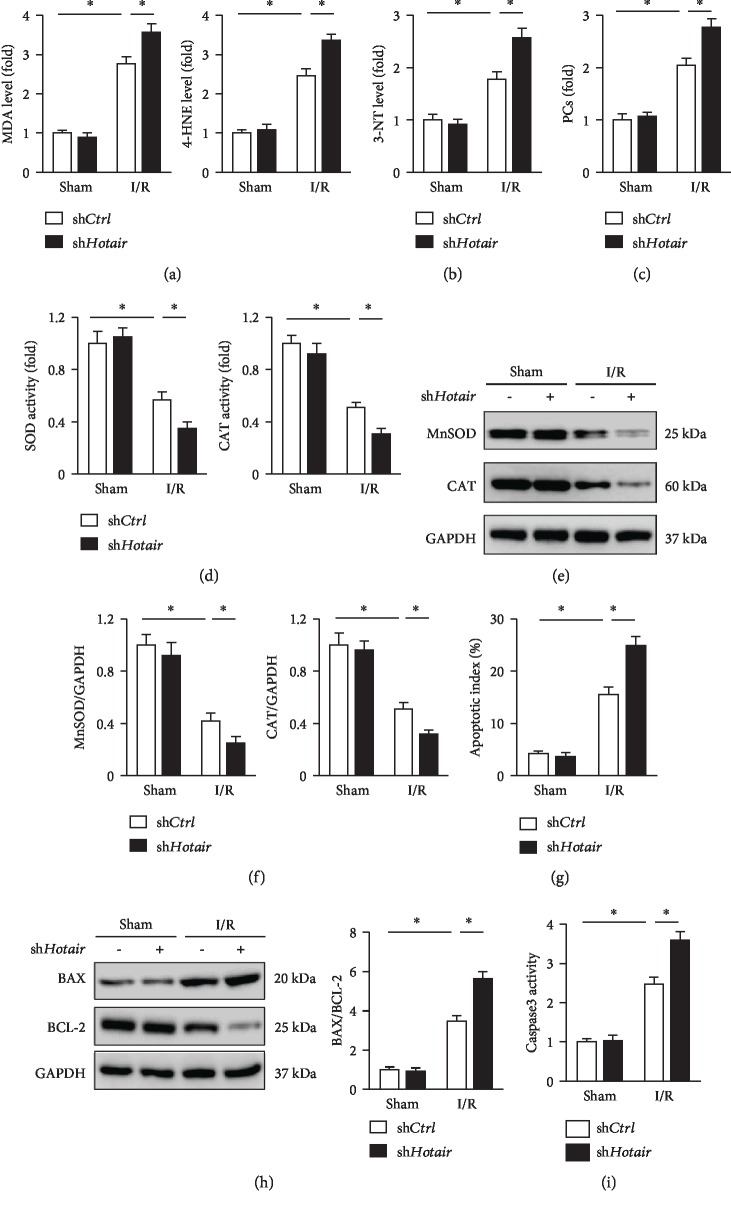
*Hotair* knockdown aggravates I/R-induced oxidative stress and cardiac myocyte apoptosis. (a–c) Myocardial malondialdehyde (MDA), 4-hydroxynonenal (4-HNE), 3-nitrotyrosine (3-NT), and protein carbonylation (PC) levels in mice with or without *Hotair* knockdown after I/R injury (*n* = 6). (d) Enzymatic activities of superoxide dismutase (SOD) and catalase (CAT) in murine hearts (*n* = 6). (e, f) MnSOD and CAT expression changes were evaluated by western blot in *Hotair*-inhibited murine hearts or their negative controls subjected to I/R (*n* = 6). (g) Cardiac myocyte apoptosis index detected by TUNEL staining (*n* = 8). (h) BAX and BCL-2 protein levels in murine hearts from the indicated groups (*n* = 6). (i) Myocardial caspase3 activity in *Hotair*-insufficient mice or their negative controls subjected to I/R surgery (*n* = 8). Data are presented as mean ± SD. ^∗^*P* < 0.05 versus the matched group.

Previous studies confirmed that oxidative stress and subsequent cardiac myocyte apoptosis play central roles in the initiation and progression of myocardial I/R injury [9, 35]. We then assessed the role of *Hotair* in I/R-induced oxidative stress and cardiac myocyte apoptosis. As shown in Figure 3(a), lipid peroxidation was enhanced in mice subjected to I/R injury, which was further aggravated by *Hotair* knockdown, as confirmed by the increased levels of MDA and 4-HNE. Protein oxidative damage is another key event for oxidative stress that contributes to I/R-induced myocardial injury. Correspondingly, we found that myocardial 3-NT and PCs were upregulated in mice assigned to I/R injury, which were notably augmented in *Hotair*-insufficient mice (Figures 3(b) and 3(c)). Superoxide-H_2_O_2_ have been identified as the key component of ROS and contributed to the development of I/R-induced cardiac dysfunction [43, 44]. MnSOD is the key enzyme to catalyze the conversion of superoxide anion to H_2_O_2_, which were then scavenged by the CAT. We thus detected MnSOD and CAT expressions and activities in I/R-injured murine hearts. In line with previous studies, the activities of SOD and CAT in murine hearts were notably suppressed by I/R surgery, and *Hotair* knockdown further decreased the capacity of endogenous cellular antioxidant defenses (Figure 3(d)). Further detection about the expressions of MnSOD and CAT verified that *Hotair* knockdown promoted oxidative stress induced by I/R operation (Figures 3(e) and 3(f)). ROS is primarily generated by NOX, among which NOX2 is the key isoform with the myocardium. We therefore examined NOX2 expression and observed that *Hotair* knockdown made no alteration on NOX2 expression in response to myocardial I/R injury ([Supplementary-material supplementary-material-1]). Unstrained oxidative stress directly induces oxidative damage to biological macromolecules and causes cell apoptosis. Accordingly, I/R surgery caused increased cell apoptosis in the myocardium, which was further enhanced in *Hotair*-insufficient mice (Figure 3(g)). Besides, western blot analysis revealed that mice with *Hotair* knockdown exhibited markedly elevated BAX/BCL-2 in comparison with the control mice (Figure 3(h)). Consistent with the above data, we found that caspase3 activity was markedly increased in murine hearts after *Hotair* knockdown in response to I/R insult (Figure 3(i)).

In line with the functional phenotype in *Hotair*-overexpressed mice, oxidative stress was drastically alleviated by *Hotair* overexpression in response to I/R operation, as indicated by the decreased myocardial MDA, 4-HNE, 3-NT, and PCs ([Supplementary-material supplementary-material-1]). On the other hand, I/R-triggered inhibition on endogenous antioxidants was also derepressed in *Hotair*-overexpressed murine hearts, as revealed by the increased activities and expressions of MnSOD and CAT ([Supplementary-material supplementary-material-1]). Western blot analysis also demonstrated that *Hotair* overexpression attenuated I/R-induced upregulation of BAX and downregulation of BCL-2, and decreased BAX/BCL-2 ([Supplementary-material supplementary-material-1]). In addition, we confirmed that the increased caspase3 activity and the induction of cell apoptosis were both improved in *Hotair*-overexpressed hearts ([Supplementary-material supplementary-material-1]). Therefore, we concluded that *Hotair* knockdown exacerbated, whereas *Hotair* overexpression ameliorated I/R-induced oxidative stress and cardiac myocyte apoptosis in murine hearts.

### 3.4. Hotair Prevents Oxidative Stress and Cardiac Myocyte Apoptosis in Response to H/R In Vitro

**Figure 4:**
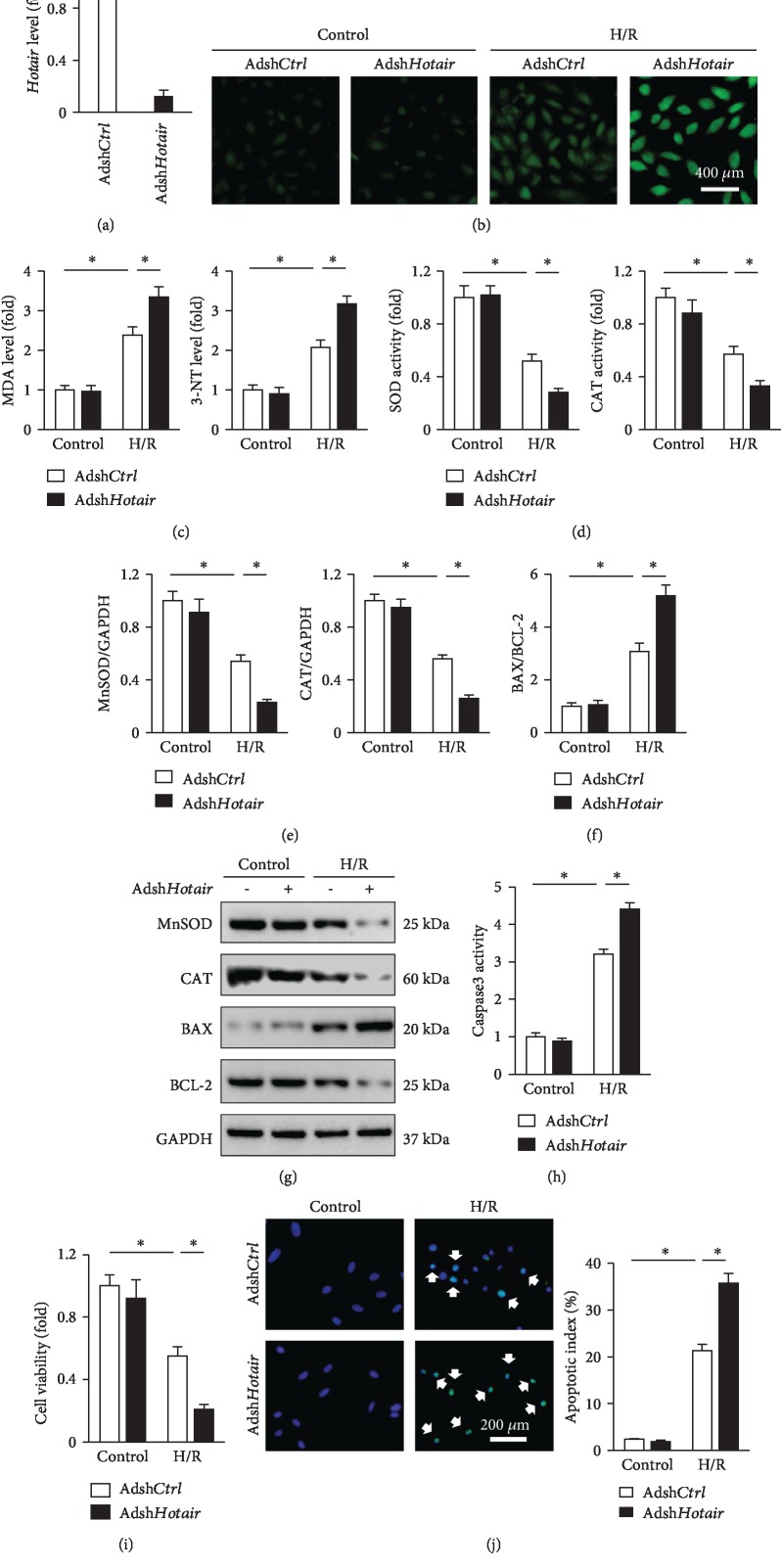
*Hotair* knockdown exacerbates oxidative stress and cardiac myocyte apoptosis in response to H/R in vitro. (a) *Hotair* expression in H9c2 cells infected with Adsh*Ctrl* or Adsh*Hotair* (*n* = 6). (b) Representative images of DCFH-DA in H9c2 cells with or without *Hotair* knockdown after H/R stimulation (*n* = 8). (c) MDA and 3-NT levels in H9c2 cells (*n* = 6). (d) Enzymatic activities of SOD and CAT in H9c2 cells (*n* = 6). (e–g) MnSOD, CAT, BAX, and BCL-2 expression changes in H/R-stimulated H9c2 cells with or without Adsh*Hotair* treatment (*n* = 6). (h) Caspase3 activity in H9c2 cells (*n* = 6). (i) Cell viability assessed by the CCK-8 assay (*n* = 6). (j) TUNEL staining and the statistical result in H9c2 cells; white arrows indicate TUNEL-positive nuclei (*n* = 8). Data are presented as mean ± SD. ^∗^*P* < 0.05 versus the matched group.

To further verify the role of *Hotair* in vitro, we knocked down the endogenous *Hotair* expression in H9c2 cells and then the cells were subjected to H/R stimulation. The efficiency was clarified by the PCR data (Figure 4(a)). DCFH-DA staining suggested that *Hotair* knockdown significantly increased ROS generation after H/R (Figure 4(b)). Correspondingly, H9c2 cells infected with Adsh*Hotair* exhibited enhanced MDA and 3-NT production (Figure 4(c)). The antioxidant enzymes, SOD, and CAT activities together with protein levels were further decreased in *Hotair*-insufficient cells (Figures 4(d) and 4(e)). Conversely, BAX/BCL-2 and caspase3 activity were notably increased in H9c2 cells after *Hotair* knockdown in the presence of H/R stimulation (Figures 4(f)–4(h)). Consistent with the molecular alteration, we observed that *Hotair* silence notably decreased cell viability and augmented H/R-induced cardiac myocyte apoptosis (Figures 4(i) and 4(j)).

H9c2 cells were also infected with Ad*Hotair* to overexpress *Hotair* before H/R stimulation, which caused ~6.9 fold increase of *Hotair* expression ([Supplementary-material supplementary-material-1]). As shown in [Supplementary-material supplementary-material-1], H/R stimulation led to increased ROS generation and caused oxidative stress in Ad*Ctrl*-infected cells, which were markedly attenuated in cells with *Hotair* overexpression, as shown by the DCFH-DA staining and decreased MDA, 3-NT levels. The decreased activities and protein levels of SOD and CAT were both preserved in Ad*Hotair*-treated H9c2 cells compared with that infected with Ad*Ctrl* ([Supplementary-material supplementary-material-1]). Protein analysis also revealed that the upregulation of BAX/BCL-2 was notably attenuated in cells with *Hotair* overexpression ([Supplementary-material supplementary-material-1]). Further detection confirmed that *Hotair* overexpression improved H/R-induced cardiac myocyte apoptosis, as evidenced by the decreased caspase3 activity and increased cell viability ([Supplementary-material supplementary-material-1]). The in vitro experiments clearly validated a beneficial role of *Hotair* in the pathogenesis of H/R-induced oxidative stress and cardiac myocyte apoptosis.

### 3.5. Hotair Alleviates H/R-Induced Oxidative Stress and Cardiac Myocyte Apoptosis via Activating AMPK*α*

**Figure 5:**
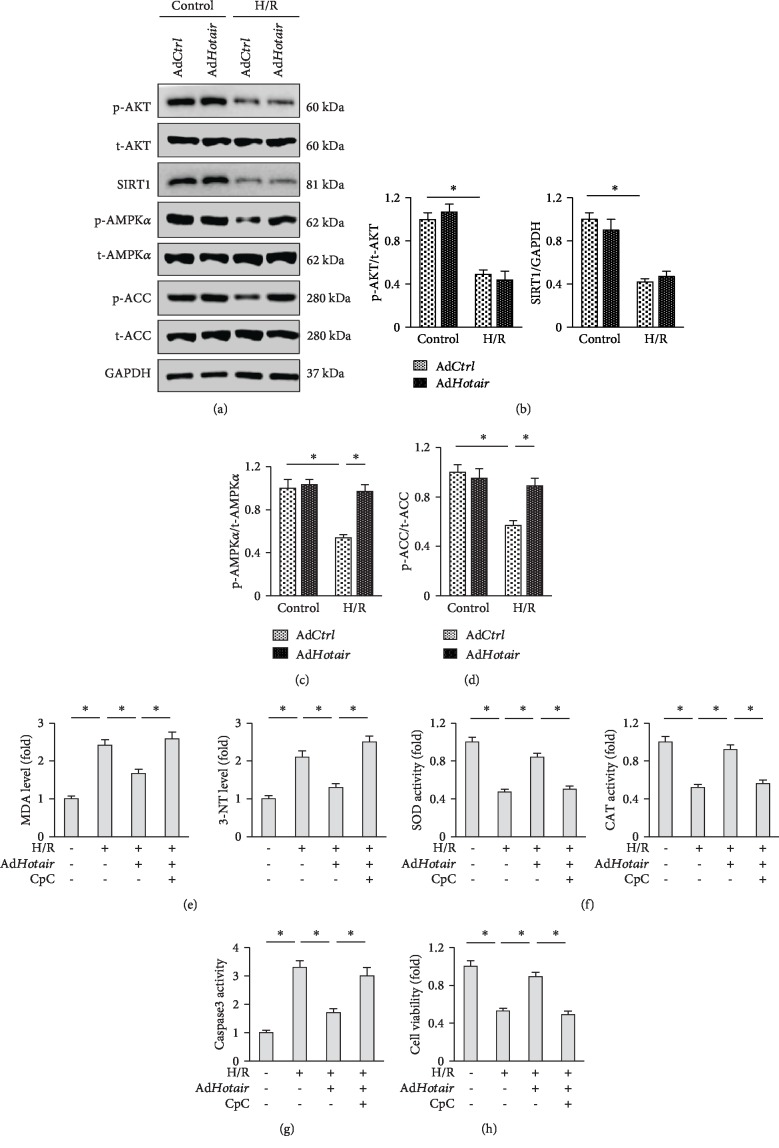
*Hotair* alleviates H/R-induced oxidative stress and cardiac myocyte apoptosis via activating AMPK*α*. (a–d) Representative western blot images and the quantitative data (*n* = 6). (e) MDA and 3-NT levels in H9c2 cells (*n* = 6). (f) Enzymatic activities of SOD and CAT in H9c2 cells (*n* = 6). (g) Caspase3 activity in H9c2 cells (*n* = 6). (h) Cell viability assessed by the CCK-8 assay (*n* = 6). Data are presented as mean ± SD. ^∗^*P* < 0.05 versus the matched group.

Next, we investigated the molecular mechanisms underlying the protective effects of *Hotair* in vitro. Given the capacity of *Hotair* to regulate AKT and the involvement of AKT pathway in I/R-induced myocardial injury, we first detected the level of AKT phosphorylation in cultured H9c2 cells. However, despite the markedly inhibition on AKT activation induced by H/R stimulation, we observed no significant alterations in AKT phosphorylated levels by *Hotair* overexpression (Figures 5(a) and 5(b)). We then investigated the potential role of SIRT1, which was well identified as the downstream target of *Hotair* in heart. Western blot result showed that Ad*Hotair* did not affect SIRT1 expression in response to H/R stimulation in vitro (Figures 5(a) and 5(b)). Inspiringly, we observed that H/R-triggered inhibition of AMPK*α* pathway was prevented in Ad*Hotair*-infected H9c2 cells, as verified by the increased phosphorylation of AMPK*α* and ACC (Figures 5(c) and 5(d)). To address whether AMPK*α* activation was essential for Ad*Hotair*-mediated beneficial effects in vitro, we co-treated H9c2 cells with CpC to inhibit AMPK*α*. The data showed that Ad*Hotair*-mediated inhibition on MDA and 3-NT production was blunted in cells with AMPK*α* suppression (Figure 5(e)). Consistently, *Hotair* overexpression significantly increased the activities of SOD and CAT in H9c2 cells, but not in that pretreated with CpC (Figure 5(f)). Notably, CpC incubation also largely blunted the protective effect of Ad*Hotair* on cardiac myocyte apoptosis, which was confirmed by the increased caspase3 activity and decreased cell viability (Figures 5(g) and 5(h)). Taken together, these data suggested that AMPK*α* activation appeared to be essential for *Hotair* overexpression-mediated protective effects in vitro.

### 3.6. Hotair Overexpression Loses Its Beneficial Effects in AMPK*α*-Inhibited Mice

**Figure 6:**
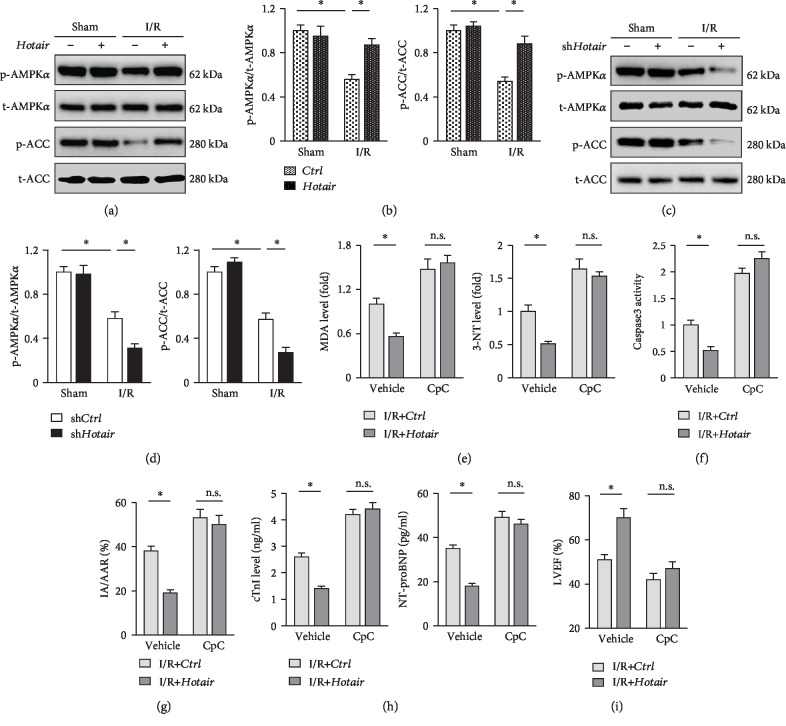
*Hotair* overexpression loses its beneficial effects in AMPK*α*-inhibited mice. (a, b) Representative western blot images and the quantitative data in I/R-treated murine hearts with or without *Hotair* overexpression (*n* = 6). (c, d) Representative western blot images and the quantitative data in murine hearts subjected to I/R surgery (*n* = 6). (e) MDA and 3-NT levels in murine hearts (*n* = 6). (f) Caspase3 activity in murine hearts (*n* = 6). (g) Morphometric analysis of IA in I/R-treated murine hearts (*n* = 8). (h) Serum biomarkers related to cardiac injury in mice (*n* = 8). (i) Echocardiographic parameters of murine hearts (*n* = 8). Data are presented as mean ± SD. ^∗^*P* < 0.05 versus the matched group.

Consistent with the in vitro data, we found that *Hotair* overexpression significantly preserved AMPK*α* activation in murine hearts after I/R injury, and conversely, AMPK*α* and ACC phosphorylation were further decreased in *Hotair*-inhibited murine hearts (Figures 6(a)–6(d)). As shown in Figure 6(e), *Hotair* overexpression markedly decreased MDA and 3-NT production in murine hearts after I/R surgery but had no protective effects in that treated with CpC. In addition, *Hotair* overexpression-mediated inhibitory effect on caspase3 activity was also abrogated by AMPK*α* inhibition (Figure 6(f)). Accordingly, we observed that the improvement on ischemia area and myocardial injury provided by *Hotair* overexpression was retarded with AMPK*α* suppression (Figures 6(g) and 6(h)). Echocardiographic measurements showed that *Hotair* overexpression caused a significant alleviation on cardiac contractile dysfunction, reflected by the increased LVEF, yet this was lost in mice with AMPK*α* suppression (Figure 6(i)). Therefore, it could be reasonably deduced that AMPK*α* activation was responsible for *Hotair* overexpression-mediated beneficial effects on myocardial I/R injury.

### 3.7. EZH2/miR-451/Cab39 Axis Is Involved in AMPK*α* Activation Caused by Hotair

**Figure 7:**
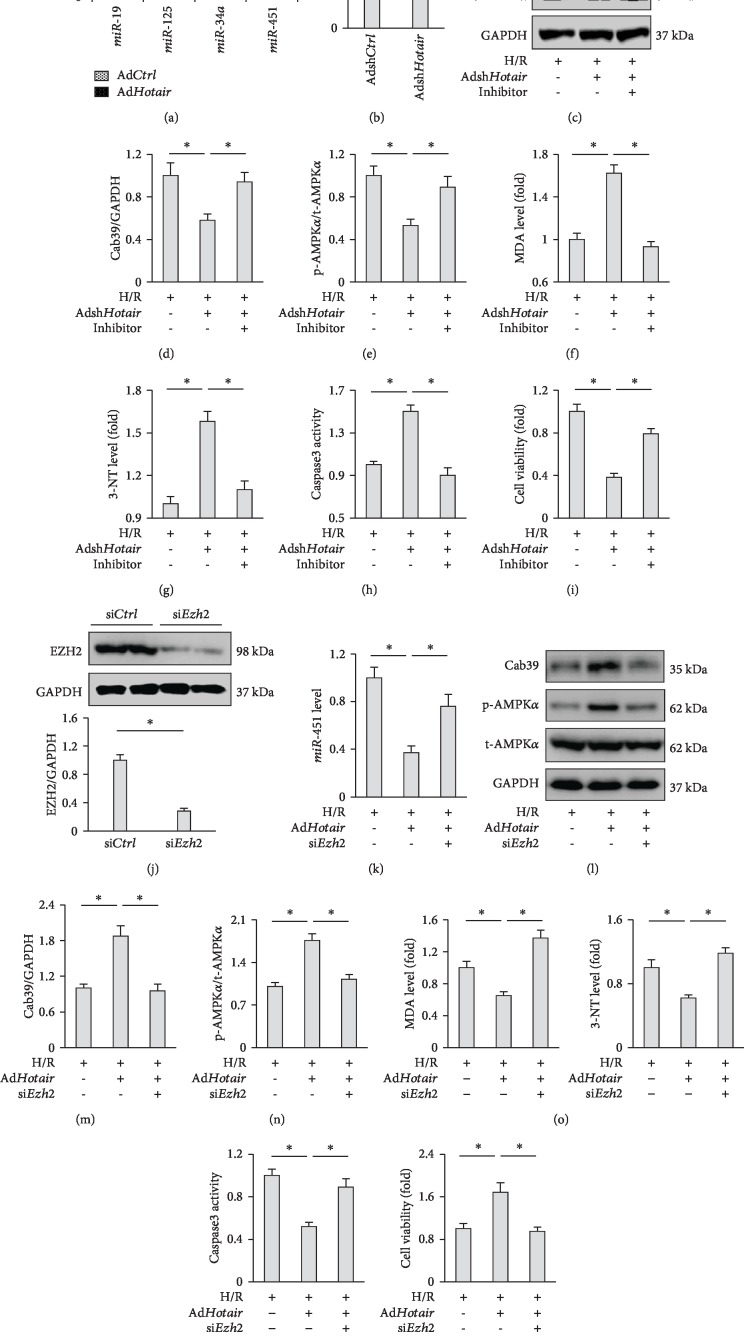
The EZH2/*miR-451*/Cab39 axis is involved in AMPK*α* activation caused by *Hotair*. (a) Expression of microRNAs (miRs) in H9c2 cells subjected to H/R injury with or without Ad*Hotair* incubation (*n* = 6). (b) The level of *miR-451* in H9c2 cells with or without *Hotair* knockdown after H/R stimulation (*n* = 6). (c–e) Representative western blot images and the quantitative data (*n* = 6). (f, g) MDA and 3-NT levels in H9c2 cells (*n* = 6). (h) Caspase3 activity in H9c2 cells (*n* = 6). (i) Cell viability assessed by the CCK-8 assay (*n* = 6). (j) The efficiency of si*Ezh2* determined by western blot in H9c2 cells (*n* = 6). (k) The level of *miR-451* in H9c2 cells in the indicated groups after H/R stimulation (*n* = 6). (l–n) Representative western blot images and the quantitative data (*n* = 6). (o) MDA and 3-NT levels in H9c2 cells (*n* = 6). (p) Caspase3 activity in H9c2 cells (*n* = 6). (q) Cell viability assessed by the CCK-8 assay (*n* = 6). Data are presented as mean ± SD. ^∗^*P* < 0.05 versus the matched group.

We then sought to determine the possible way through which *Hotair* activated AMPK*α*. Previous studies determined that lncRNAs can act on miRNA to regulate protein-coding gene expression, and *Hotair* was involved in various pathophysiological processes via negatively regulating miRNAs [45]. We then detected the level of *miR-19*, *miR-125*, and *miR-34a* in H9c2 cells, which have been verified to mediate the regulatory effects of *Hotair* on cardiac function [24, 25, 46]. As shown in Figure 7(a), we found that *Hotair* overexpression did not affect the level of *miR-19*, *miR-125*, and *miR-34a* in H/R-stimulated H9c2 cells yet markedly decreased *miR-451* expression in H9c2 cells after H/R stimulation. And *Hotair* silence notably increased *miR-451* expression in H/R-stimulated H9c2 cells (Figure 7(b)). Cab39 was shown to form a heterotrimeric complex with ste20-related adaptor (STRAD) and served as a scaffold protein for liver kinase B1 (LKB1), an upstream kinase of AMPK*α*, to stabilize its activity [47]. It is well-accepted that *miR-451* plays an essential role in modulating AMPK*α* pathway via directly targeting Cab39; we thus detected Cab39 protein expression in sh*Hotair*-treated murine hearts [48]. As shown in [Supplementary-material supplementary-material-1], we found that Cab39 was downregulated in *Hotair*-deficient murine hearts after I/R surgery. To notify the involvement of *miR-451* in *Hotair*-mediated regulation on Cab39/AMPK*α*, we treated H9c2 cells with *miR-451* inhibitor. As expected, we found that Adsh*Hotair*-mediated inhibition on Cab39 mRNA and protein was markedly reversed by the *miR-451* inhibitor (Figures 7(c) and 7(d), [Supplementary-material supplementary-material-1]). Correspondingly, AMPK*α* dephosphorylation and the augmented oxidative stress as well as cardiac myocyte apoptosis induced by *Hotair* knockdown were all abolished in the presence of a *miR-451* inhibitor, as confirmed by the decreased MDA, 3-NT level, caspase3 activity, and increased cell viability (Figures 7(e)–7(i)).

We finally aimed to elucidate the regulatory mechanism between *Hotair* and *miR-451* in the context of I/R injury. Previous data showed that *Hotair* functioned as a molecular sponge on *miR-19*, *miR-125*, and *miR-34a* to regulate cardiac pathophysiology; however, we observed no alteration of these miRNAs in the present study, which implied the existence of a distinct modulation. Emerging evidences suggested that *Hotair* could recruit polycomb repressive complex 2 (PRC2) and lead to epigenetic silence of target genes. Furthermore, Cheng et al. recently clarified that *Hotair* suppressed *miR-122* expression in hepatocellular carcinoma by an epigenetic mechanism [49]. EZH2, an important component of PRC2, was reported to be responsible for the downregulation of many miRNAs. We therefore tried to ascertain whether EZH2 contributed to *Hotair*-mediated *miR-451* inhibition after H/R stimulation. As shown in Figures 7(j) and 7(k), Ad*Hotair*-stimulated downregulation on *miR-451* was abolished by EZH2 silence. Subsequently, Cab39/AMPK*α* activation in Ad*Hotair*-infected cells was suppressed after EZH2 knockdown (Figures 7(l)–7(n)). In line with the molecular alteration, we found that *Hotair* overexpression-elicited beneficial effect on H/R-induced oxidative damage and cardiac myocyte apoptosis was completely retarded in si*Ezh2*-infected cells (Figures 7(o)–7(q)). Thus, we concluded that EZH2/*miR-451*/Cab39 axis was involved in AMPK*α* activation caused by *Hotair*.

## 4. Discussion

In the present study, we found that *Hotair* was upregulated in response to I/R injury via a BRD4-dependent manner. Cardiac-restricted knockdown of *Hotair* exacerbated, whereas *Hotair* overexpression prevented I/R-induced oxidative stress, cardiac myocyte apoptosis, and cardiac dysfunction. Further detection showed that *Hotair* exerted the protective effects via regulating EZH2/*miR-451*/Cab39/AMPK*α* axis. Taken together, our preclinical studies identified *Hotair* as a potential therapeutic target for treating myocardial I/R injury.

Ischemic heart disease causes great morbidity and mortality worldwide that results in tremendous burden to individuals, families, and the whole society. Early restoration of the blood supply based either on pharmacological thrombolysis or on PCI is identified as the cornerstone in coronary heart disease therapy. However, reperfusion itself could cause a second wave of insult to the ischemic myocardium, which has been estimated to contribute to about half of the overall functional loss of the infarcted heart [1–3]. So far, there is no effective therapeutic regimen against myocardial I/R injury. Intriguingly, our data suggested that lncRNA *Hotair* in cardiac myocytes was instrumental for the heart to counteract I/R-induced injury and dysfunction. Numerous studies have been performed to explore the role of *Hotair* in cardiovascular diseases, including the ischemic damage, yet the exact role of *Hotair* in oxidative stress and cardiac myocyte apoptosis during I/R injury remains delusive. Lu et al. previously observed that *Hotair* overexpression promoted the synthesis and release of inflammatory cytokines from hypoxia-induced H9c2 cells, which is in line with a previous study found that high *Hotair* level promoted the onset of cerebral infarct and dysfunction [27, 50]. In contrast, data from other studies indicated that *Hotair* overexpression suppressed hypoxia- or oxidative stress-induced cardiac myocyte injury [46, 51]. Even so, the role of *Hotair* in ischemic hearts cannot be transposed to the myocardial I/R injury condition. Besides, several studies identified *Hotair* expression was decreased in myocytes exposed to hypoxia/oxidative damage and in the serum from AMI patients [46, 51]. Yet, we herein found that *Hotair* was upregulated in murine hearts subjected to I/R surgery and H9c2 cells exposed to H/R stimulation. The different expression pattern during myocardial ischemia and I/R implied a distinct role for *Hotair* in the progression of myocardial I/R injury. In the present study, gain- and loss-of-function studies clearly corroborated *Hotair* overexpression has a beneficial effect on I/R-triggered oxidative stress, apoptosis, and cardiac dysfunction in vivo and in vitro. Previous studies implied that the same intervention might cause diametrically opposite outcome in different disease models, and more importantly, a study from Zhai et al. defined differential roles of GSK3*β* in myocardial ischemia and I/R injury [52–54]. Matsui et al. further proved that the pathophysiologic mechanism might be distinctly different during myocardial ischemia and I/R [55]. Collectively, our data elucidated the distinctly beneficial role of *Hotair* in myocardial I/R injury.

Accumulation of ROS within the myocardium has been verified as the central mechanism of myocardial I/R injury [9]. Nutrient deficit and oxygen deprivation due to coronary artery occlusion elicit distinct alterations in intracellular signaling axis and metabolic status. As a consequence, free fatty acid oxidation is inhibited and glycolysis becomes the predominant means of energy production. However, the sudden influx of nutrients together with the restoration of oxygen supply reconstructs the oxidative phosphorylation in cardiac myocytes for more efficient ATP generation, which meanwhile leads to ROS overproduction via the disrupted electron transport chain [56]. Fulminant oxygen free radicals directly modify proteins via posttranslational methods, including nitration and carbonylation, which subsequently destroy protein functions and cause cell apoptosis [9]. The abnormal expression of lncRNAs has been identified to be involved in oxidative stress and cell apoptosis and was responsible for the occurrence of myocardial I/R injury. Li et al. recently found that lncRNA H19 functioned as a competing endogenous RNA of *miR-877-3p* and alleviated cardiac myocyte apoptosis and myocardial I/R damage [57]. Su and colleagues proved that lncRNA TUG1 inhibition upregulated *miR-142-3p* and ameliorated myocardial injury in I/R-stimulated hearts [21]. Results from Wang et al. also revealed that lncRNA NRF directly repressed *miR-873* expression and regulated cardiac myocyte necrosis and myocardial I/R injury in mice [58]. In the current study, we clarified that lncRNA *Hotair* overexpression prevented oxidative stress, cardiac myocyte apoptosis, and cardiac malfunction after I/R surgery via activating AMPK*α*. AMPK*α* is commonly regarded as the endogenous energy sensor and maintains energy homoeostasis via regulating cellular ATP levels [10]. In addition to sensing energy deficit, AMPK*α* also plays essential roles in the pathogenesis of heart diseases. Zhang et al. found that activation of AMPK*α* prevented cardiac fibrosis following long-term pressure overload [12]. Besides, a recent study also confirmed that AMPK*α* activation notably alleviated oxidative stress and cardiac myocyte apoptosis in doxorubicin-treated murine hearts, thereby preventing doxorubicin-induced cardiac impairment [8]. Moreover, Wang et al. observed that mice with cardiac-specific inhibition of AMPK*α* exhibited increased oxidative stress and cardiac myocyte apoptosis in response to myocardial I/R injury, indicating AMPK*α* as a cardioprotective factor against I/R-induced cardiac injury and malfunction [15]. Our current data suggested that AMPK*α* was responsible for the protective effects of *Hotair* against myocardial I/R injury.

We finally investigated the possible mechanism through which *Hotair* activated AMPK*α*. Previous studies determined that lncRNAs can act on miRNAs to regulate protein-coding gene expression and *Hotair* has been shown to regulate cardiac pathophysiology via *miR-19*, *miR-125*, and *miR-34a*. Unexpectedly, we found that *Hotair* manipulation did not affect these miRNAs in the context of myocardial I/R injury but caused *miR-451* alteration. Cab39 was shown to form a heterotrimeric complex with STRAD and contributed to the stabilization of LKB1 activity [47]. Previous studies proved that *miR-451* inactivated AMPK*α* pathway via directly targeting Cab39 [48]. In line with these data, we observed that Cab39 expression was decreased in *Hotair*-deficient cardiac myocytes or heart samples, which was prevented by the treatment of a *miR-451* inhibitor. Generally, lncRNAs functioned as a molecular sponge of miRNAs, and the modulation of *Hotair* on *miR-19*, *miR-125*, and *miR-34a* was also related to the sponge function. Intensive studies showed that individual lncRNAs can also act as the interface between DNA and specific chromatin remodeling activities. Data from Chang lab previously determined that *Hotair* overexpression in epithelial cancer cells could trigger genome-wide retargeting of PRC2 within genomic loci [22]. Further detection revealed that *Hotair* bound to both PRC2 and lysine-specific demethylase 1 (LSD1) complex, which subsequently resulted in a silent chromatin state and transcriptional repression [59]. EZH2, a histone 3 lysine 27 (H3K27) methyltransferase, is an important component of PRC2 and plays critical roles in regulating miRNAs silence. In accordance with previous studies, we found *Hotair* inhibited *miR-451* via EZH2, and *Ezh2* deficiency abrogated the inhibitory effect of *Hotair* in vitro.

**Figure 8:**
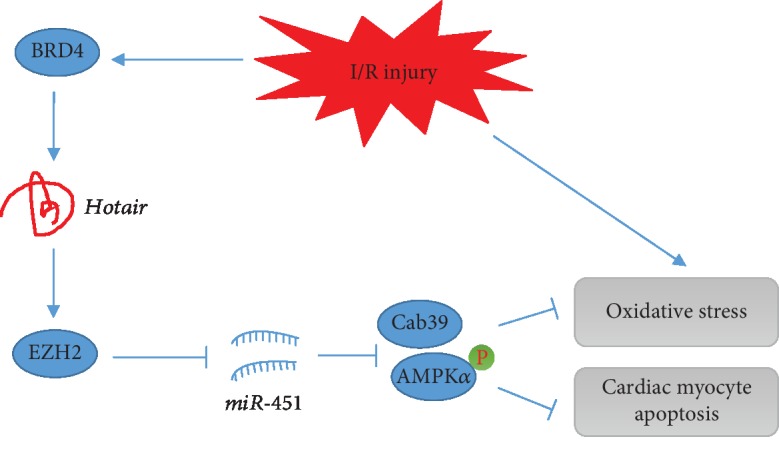
The proposed model of the role of Hotair in myocardial I/R injury. *Hotair* was upregulated in response to I/R stimuli. *Hotair* exerted a protective effect on oxidative stress and cardiac myocyte apoptosis via regulating the enhancer of zeste homolog 2/microRNA-451/calcium-binding protein 39/AMP-activated protein kinase alpha (EZH2/*miR-451*/Cab39/AMPK*α*) axis.

In summary, we provide the evidence that endogenous lncRNA *Hotair* is an essential negative regulator for the progression of myocardial I/R injury, which is dependent on AMPK*α* activation via the EZH2/*miR-451*/Cab39 axis (Figure 8). Therefore, targeting *Hotair* might provide novel insight into developing effective therapeutic strategies for the treatment of myocardial I/R injury.

## Data Availability

The data that support the findings of this study are available from the corresponding author upon reasonable request.

## Supplementary Materials

Supplementary Materials Figure S1: Hotair overexpression protected against I/R-induced oxidative stress and cardiac myocyte apoptosis. Figure S2: Hotair overexpression prevented oxidative stress and cardiac myocyte apoptosis in response to H/R in vitro. Figure S3: the expression of Cab39 in vivo and in vitro. A. Cab39 protein level in Hotair-deficient murine hearts after I/R injury (*n* = 6). B. Cab39 mRNA level in H/R-stimulated H9c2 cells in the indicated groups (*n* = 6). Data are presented as mean ± SD (^∗^*P* < 0.05 versus the matched group).
