# Supplementary material for: The Long Noncoding RNA Hotair Regulates Oxidative Stress and Cardiac Myocyte Apoptosis during Ischemia-Reperfusion Injury
Source: Oxid Med Cell Longev. 2020 Mar 12;2020:1645249. doi: 10.1155/2020/1645249 (PMC7091551; doi:10.1155/2020/1645249)
Supplement: Supplementary Materials — Figure S1: Hotair overexpression protected against I/R-induced oxidative stress and cardiac myocyte apoptosis. Figure S2: Hotair overexpression prevented oxidative stress and cardiac myocyte apoptosis in response to H/R in vitro. Figure S3: the expression of Cab39 in vivo and in vitro. A. Cab39 protein level in Hotair-deficient murine hearts after I/R injury (n = 6). B. Cab39 mRNA level in H/R-stimulated H9c2 cells in the indicated groups (n = 6). Data are presented as mean ± SD (∗P < 0.05 versus the matched group). [file 1645249.f1.docx]

**Data supplement**

**The long noncoding RNA Hotair regulates oxidative stress and cardiac myocyte apoptosis during ischemia-reperfusion injury**

Running title: *Hotair* alleviates myocardial I/R injury

Kai Meng^1,*^, Jiao Jiao^1,*^, Rui-Rui Zhu^1^, Bo-Yuan Wang^1^, Xiao-Bo Mao^1^, Yu-Cheng Zhong^1^, Zheng-Feng Zhu^1^, Kun-Wu Yu^1^, Yan Ding^1^, Wen-Bin Xu^1^, Jian Yu^1^, Qiu-Tang Zeng^1^, Yu-Dong Peng^1,#^

^1^ Department of Cardiology, Union Hospital, Tongji Medical College, Huazhong University of Science and Technology, Wuhan 430022, China

^*^ These authors contributed equally to this work.

**^#^ Corresponding Author: Yu-Dong Peng**

Department of Cardiology,

Union Hospital, Tongji Medical College,

Huazhong University of Science and Technology,

1277 Jiefang Avenue, Wuhan 430022, China

E-mail: am-penicillin@163.com


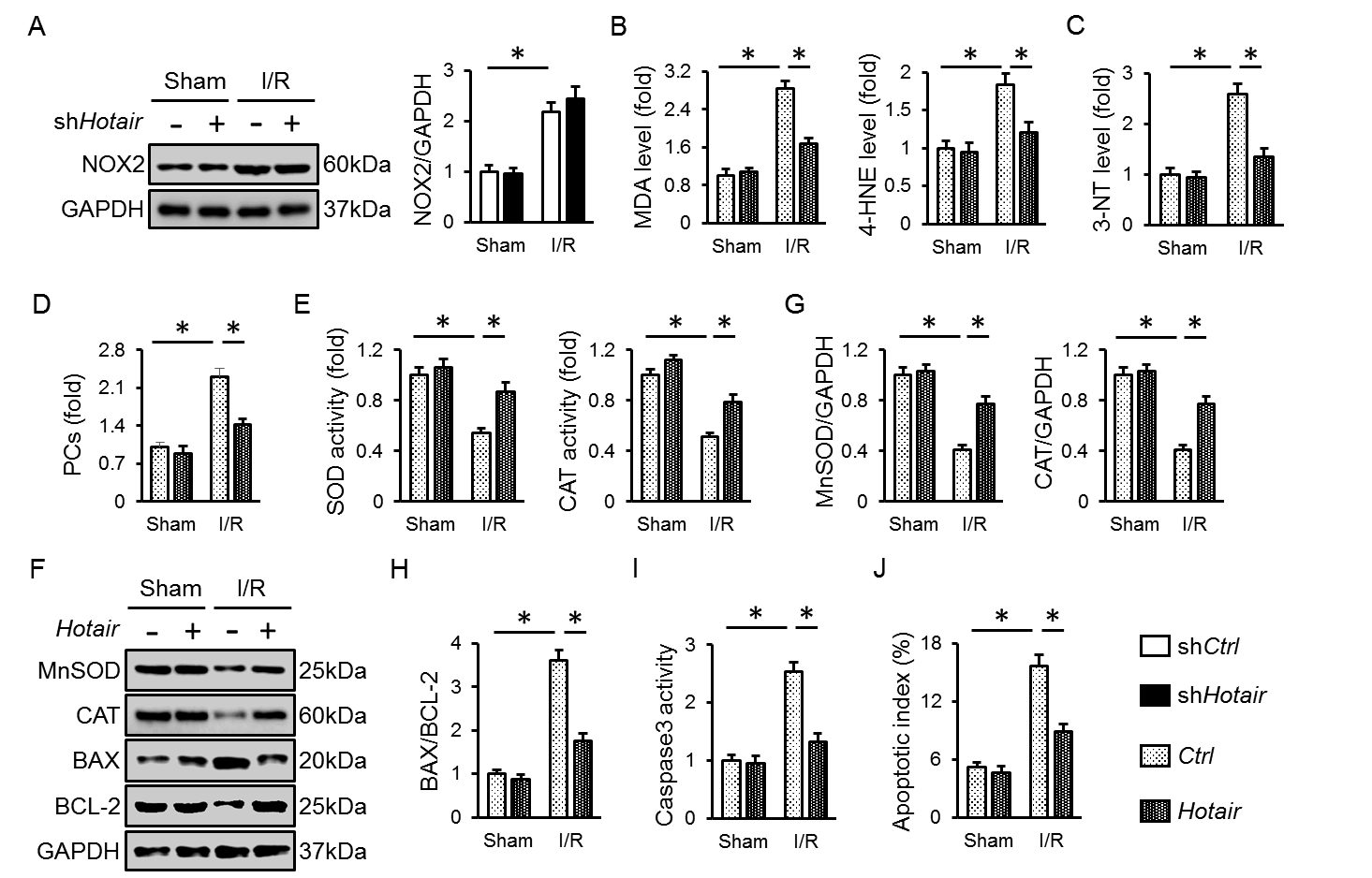


**Figure S1. *Hotair* overexpression protected against I/R-induced oxidative stress and cardiac myocyte apoptosis.** **A.** NOX2 protein levels in murine hearts with or without Hotair knockdown after I/R surgery (n=6). **B-D.** Myocardial MDA, 4-HNE, 3-NT and PCs levels in mice with or without *Hotair* overexpression after I/R injury (n=6). **E.** Enzymatic activities of SOD and CAT in murine hearts (n=6). **F-H.** MnSOD, CAT, BAX and BCL-2 expression changes were evaluated by western blot in I/R-treated murine hearts with or without AAV9-*Hotair* treatment (n=6). **I.** Myocardial caspase3 activity (n=8). **J.** Cardiac myocyte apoptosis index detected by TUNEL staining (n=8). Data were presented as mean±SD, **P*﹤0.05 versus the matched group.


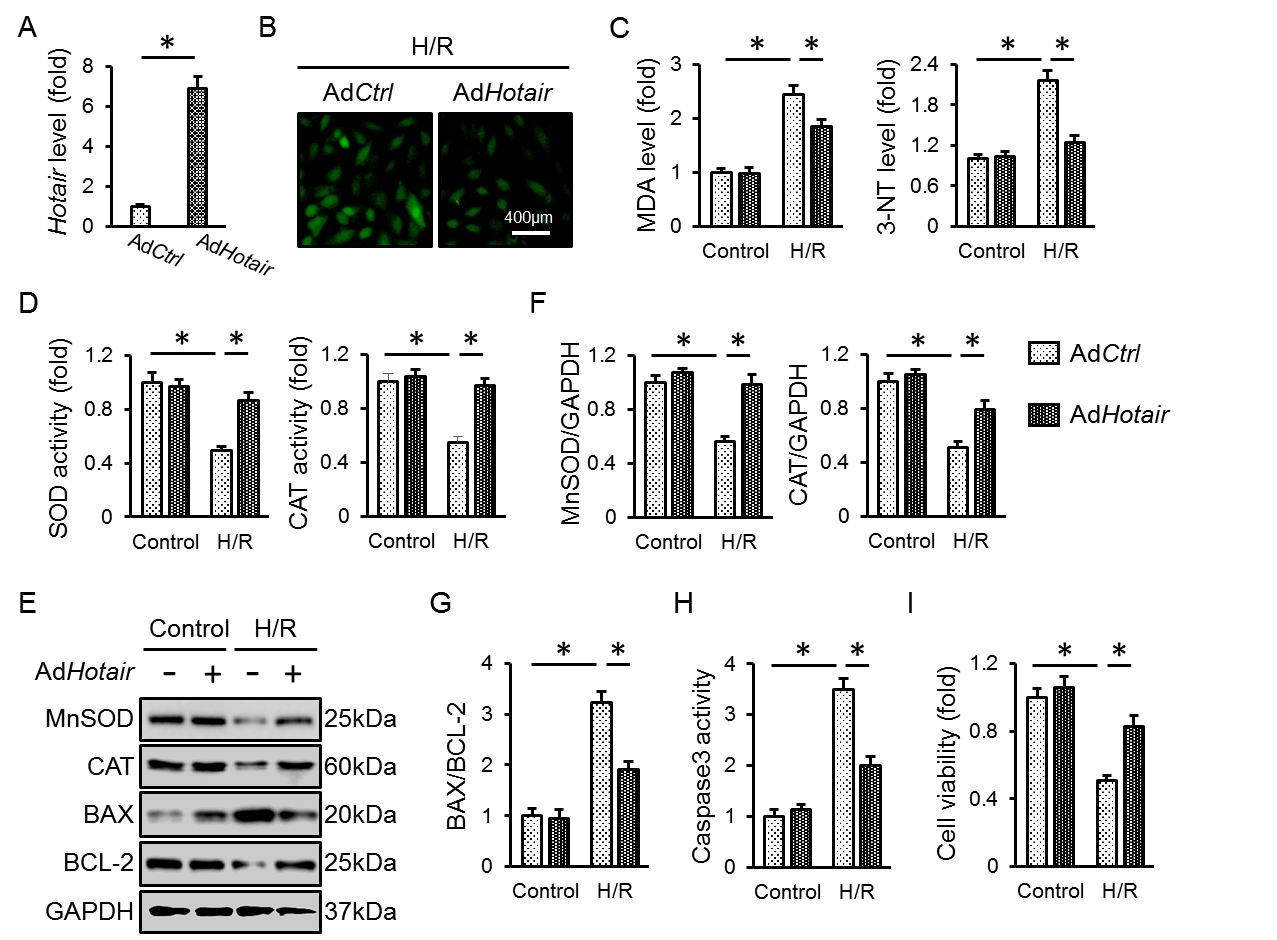


**Figure S2. *Hotair* overexpression prevented oxidative stress and cardiac myocyte apoptosis in response to H/R in vitro.** **A.** *Hotair* expression in H9c2 cells infected with Ad*Ctrl* or Ad*Hotair* (n=6). **B.** Representative images of DCFH-DA in H9c2 cells with or without *Hotair* overexpression after H/R stimulation (n=8). **C.** MDA and 3-NT levels in H9c2 cells (n=6). **D.** Enzymatic activities of SOD and CAT in H9c2 cells (n=6). **E-G.** MnSOD, CAT, BAX and BCL-2 expression changes in H/R-stimulated H9c2 cells with or without Ad*Hotair* treatment (n=6). **H.** Caspase3 activity in H9c2 cells (n=6). **I.** Cell viability assessed by CCK-8 assay (n=6). Data were presented as mean±SD, **P*﹤0.05 versus the matched group.


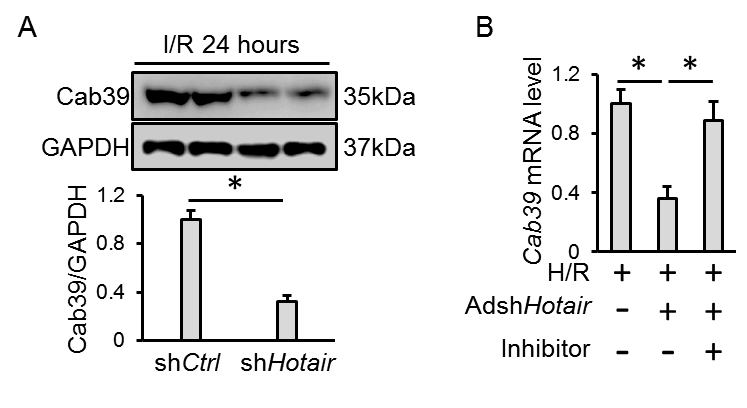


**Figure S3. The expression of Cab39 in vivo and in vitro.** **A.** Cab39 protein level in *Hotair*-deficient murine hearts after I/R injury (n=6). **B.** *Cab39* mRNA level in H/R-stimulated H9c2 cells in indicating groups (n=6). Data were presented as mean±SD, **P*﹤0.05 versus the matched group.
